# Editorial: Microbiota, antibiotic resistance, and host-microbe interactions: a comprehensive exploration of infectious disease dynamics

**DOI:** 10.3389/fcimb.2026.1899262

**Published:** 2026-06-26

**Authors:** Anis Rageh Al-Maleki, Samantha Flores-Treviño, Chia Wei Cheah, Yousri Abdelmutalab Abdelhafiz

**Affiliations:** 1 Department of Medical Microbiology, Faculty of Medicine, Universiti Malaya, Kuala Lumpur, Malaysia; 2 Department of Medical Microbiology, Faculty of Medicine and Health Sciences, Sana’a University, Sana’a, Yemen; 3 Department of Infectious Diseases, Faculty of Medicine, Autonomous University of Nuevo León, Monterrey, Mexico; 4 Department of Restorative Dentistry, Faculty of Dentistry, Universiti Malaya, Kuala Lumpur, Malaysia; 5 Faculty of Biosciences and Aquaculture, Nord University, Bodø, Norway

**Keywords:** antimicrobial resistance, host–microbe interactions, infectious disease dynamics, metagenomics, microbial dysbiosis, microbiome therapeutics, microbiota, multidrug-resistant pathogens

## Introduction

**Figure 1:**
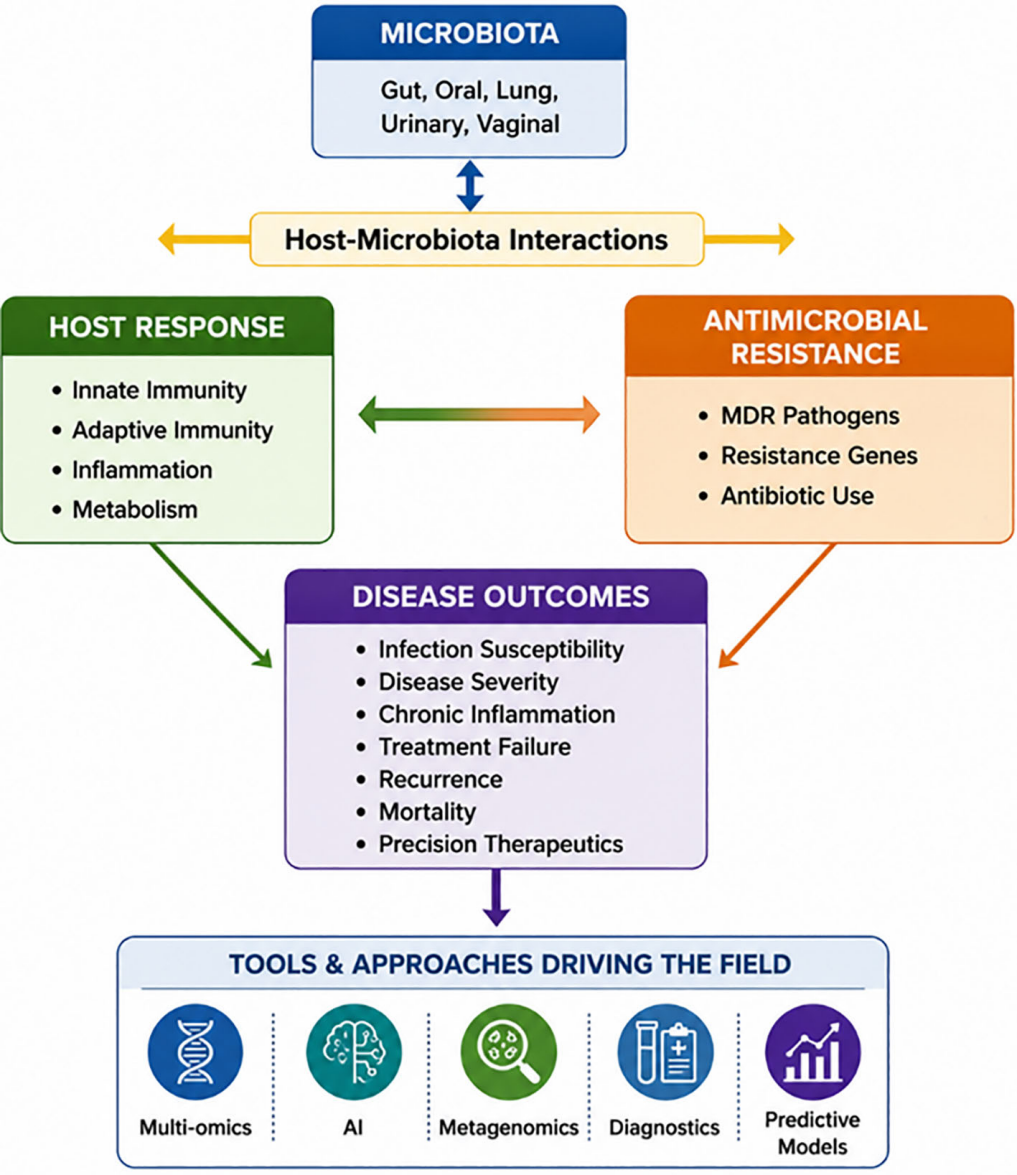
Interconnected roles of microbiota, host immunity, and antimicrobial resistance in infectious disease dynamics. Microbial communities influence host immunity, inflammation, and disease susceptibility, while host responses shape microbial composition and ecological stability. Antimicrobial resistance further alters microbial ecosystems and treatment outcomes through the emergence of multidrug-resistant pathogens, resistance genes, and antibiotic selective pressures. The interactions among these components collectively contribute to infection susceptibility, disease severity, chronic inflammation, treatment failure, recurrence, mortality, and opportunities for precision therapeutics. Emerging tools including multi-omics, artificial intelligence, metagenomics, diagnostics, and predictive models facilitate a deeper understanding of these complex relationships.

The human microbiota plays a fundamental role in maintaining physiological homeostasis, regulating immune responses, and protecting against pathogen colonization. Recent advances in high-throughput sequencing, metagenomics, multi-omics technologies, and computational biology have transformed our understanding of infectious diseases from a pathogen-centered perspective toward a systems-level framework that recognizes the dynamic interactions among microbial communities, host responses, environmental influences, and antimicrobial pressures (Lynch and Pedersen, 2016; Gilbert et al., 2018; Fan and Pedersen, 2021). Simultaneously, antimicrobial resistance (AMR) has emerged as one of the most pressing global health threats, undermining therapeutic effectiveness and increasing disease burden worldwide (Murray et al., 2022). This Research Topic, “*Microbiota, Antibiotic Resistance, and Host-Microbe Interactions: A Comprehensive Exploration of Infectious Disease Dynamics*”, brings together nineteen contributions that collectively advance our understanding of these interconnected domains and highlight emerging opportunities for diagnosis, prevention, and treatment (**Figure 1**).

A major theme emerging from this Research Topic is the influence of microbial dysbiosis on disease susceptibility and progression. Alterations in gut microbial communities were implicated in stroke-associated pneumonia, supporting the growing recognition of the gut–lung axis as an important regulator of infection susceptibility (Xiao et al.). Similarly, significant disturbances in gut and urinary microbiota were observed among patients with urolithiasis, suggesting that microbial imbalances may contribute to disease recurrence even after surgical intervention (Jia et al.). The importance of microbial ecosystem stability was further emphasized by findings describing abnormalities in the intestinal microenvironment of HIV immunological non-responders, highlighting potential links between persistent dysbiosis and impaired immune recovery (Liu and Yang).

The impact of microbiota extends beyond infectious diseases and increasingly influences chronic inflammatory and systemic conditions. The review by Liu SH et al. highlighted the regulatory role of the gut microbiota–short-chain fatty acid signaling axis in slow transit constipation and discussed emerging multi-target therapeutic strategies. Complementing these findings, Herrera-Saldivar et al. reviewed the interplay between probiotics and mast cells, emphasizing the ability of beneficial microorganisms to modulate inflammatory responses and maintain mucosal homeostasis. Likewise, Zhang et al. demonstrated the complex relationship between vulvovaginal candidiasis, vaginal microbial ecology, human papillomavirus infection, and cervical lesion development, illustrating how microbial community composition can influence disease outcomes.

Several studies further expanded our understanding of microbial products as active mediators of disease. Guo et al. demonstrated that outer membrane vesicles derived from *Porphyromonas gingivalis* induced Alzheimer’s disease-like pathological features in zebrafish, supporting growing evidence linking oral pathogens to neurodegenerative disorders. Similarly, Shibata et al. reported that urinary microbiota and bacterial membrane vesicles may contribute to chronic kidney disease-associated antimicrobial-resistant urinary tract infections. These studies highlight the increasingly recognized role of microbial-derived structures in shaping host responses and disease progression.

Antimicrobial resistance represents another central theme of this Research Topic. The retrospective cohort study by Teumezgi et al. provided important insights into mortality-associated factors and treatment outcomes among patients with multidrug-resistant tuberculosis in Eritrea. The emergence of resistant fungal pathogens was highlighted by Li J et al., who characterized a clinical *Candida auris* isolate and its antifungal resistance profile. In addition, Li X et al. provided valuable epidemiological, susceptibility, and genotypic data from 214 clinical *Nocardia* isolates, contributing to improved understanding of this increasingly important opportunistic pathogen.

Beyond individual pathogens, several studies illustrated the ecological nature of antimicrobial resistance. Analysis of lower respiratory tract microbiota during antibiotic treatment of *Pseudomonas aeruginosa* pneumonia revealed substantial microbial community alterations that may influence treatment outcomes and resistance development (Jiang et al.). Similarly, Zhao et al. demonstrated that gut-to-tumor translocation of multidrug-resistant *Klebsiella pneumoniae* can reshape the tumor microbiome and contribute to chemoresistance in pancreatic cancer. Together, these findings reinforce the concept that antimicrobial resistance should be viewed not merely as a characteristic of individual microorganisms but as a complex ecological phenomenon influenced by microbial interactions, host factors, and environmental pressures.

Advances in diagnostic technologies and pathogen detection were also prominently represented in this Research Topic. Xu et al. demonstrated the value of metagenomic next-generation sequencing in identifying *Treponema denticola*-associated lung abscesses, highlighting the utility of culture-independent diagnostics for detecting difficult-to-culture organisms. Additional insights into complex infectious processes were provided by Li Y et al., who reported a rare pulmonary mixed infection involving *Nocardia cyriacigeorgica*, *Stenotrophomonas maltophilia*, and human cytomegalovirus in an immunocompromised patient. Furthermore, Li Y et al. showed that differences in microbial composition may play an important role in pancreatic pseudocyst infections, further emphasizing the importance of microbial profiling in disease management.

The interactions between microbial communities and host responses remain a critical determinant of disease outcomes. Studies examining HIV-associated dysbiosis (Liu and Yang), vulvovaginal candidiasis (Zhang et al.), and urinary microbiota-associated infections (Shibata et al.) collectively demonstrate that microbial communities and host immune systems continuously influence one another in shaping disease trajectories. These findings reinforce the concept that infectious diseases emerge from dynamic biological networks rather than isolated host–pathogen encounters.

The Research Topic also highlights the growing role of predictive analytics, artificial intelligence, and computational biology in infectious disease research. Wang et al. developed and internally validated a nomogram for predicting the severity of community-acquired pneumonia in children, demonstrating how clinical and biological variables can be integrated to improve risk stratification and patient management. Similarly, Hu and Nie proposed a dual-hypergraph contrastive learning framework for predicting microbe–drug interactions, illustrating the increasing application of machine learning approaches in antimicrobial discovery and precision medicine.

Finally, large-scale epidemiological studies continue to provide valuable insights into changing infectious disease landscapes. Yan et al. demonstrated that the COVID-19 pandemic substantially influenced the epidemiology, clinical manifestations, and molecular characteristics of *Mycoplasma pneumoniae*, emphasizing how public health interventions and societal changes can reshape pathogen transmission dynamics.

Taken together, the studies included in this Research Topic demonstrate that microbiota, antimicrobial resistance, and host responses are interconnected components of a complex biological ecosystem. By integrating microbiome analyses, molecular epidemiology, clinical investigations, computational modeling, and translational research, these contributions collectively advance our understanding of infectious disease dynamics. Future efforts should focus on integrating multi-omics technologies, longitudinal cohort studies, artificial intelligence, and systems biology approaches to identify causal mechanisms and develop precision medicine strategies. Such advances will be essential for addressing the growing challenges posed by emerging pathogens, antimicrobial resistance, and microbiome-associated diseases.

## Future perspectives

Advances in multi-omics technologies, metagenomics, artificial intelligence, and systems biology are facilitating a more comprehensive understanding of host–microbe interactions and microbial ecosystem dynamics (Hasin et al., 2017; The Integrative HMP (iHMP) Research Network Consortium, 2019). These approaches have the potential to accelerate the development of precision medicine strategies that integrate microbiome profiles, host immune responses, and pathogen characteristics to improve disease prediction and therapeutic outcomes.
